# Sterile Seroma after Drainage of Purulent Muscle Abscess in Crohn's Disease: Two Cases

**DOI:** 10.1155/2016/1516364

**Published:** 2016-07-27

**Authors:** Natasha Shah, Lara Dakhoul, Adam Treitman, Muhammed Tabriz, Charles Berkelhammer

**Affiliations:** University of Illinois, Oak Lawn, IL 60453, USA

## Abstract

Purulent skeletal muscle abscesses can occur in Crohn's disease. We report a case of a sterile seroma complicating percutaneous drainage of a purulent skeletal muscle abscess in Crohn's ileitis. We compare and contrast this case with a similar case we published earlier. We emphasize the importance of recognition and differentiation from a septic purulent abscess.

## 1. Introduction

Purulent skeletal muscle abscesses can occur in Crohn's disease [1–3]. We have previously described what we believe to be the first reported case of a sterile seroma complicating drainage of a septic psoas muscle abscess in Crohn's disease [4]. We now describe a second similar case in which a sterile seroma developed after drainage of a purulent iliacus muscle abscess in Crohn's disease. We compare and contrast both cases and encourage physicians to be aware of the potential development of sterile seromas after drainage of purulent skeletal muscle abscesses.

## 2. Case Report

**Figure 1:**
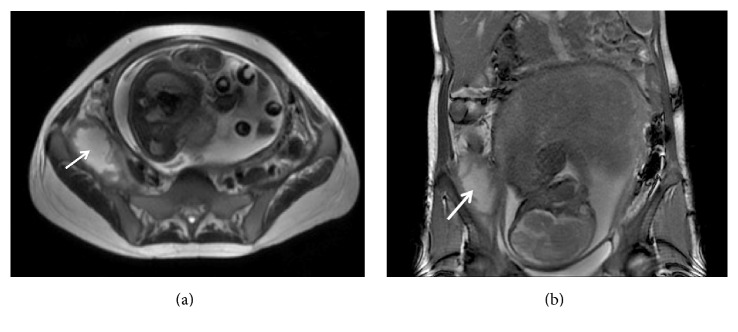
MRI showing coronal (a) and axial (b) views of right iliacus septic muscle abscess complication of Crohn's ileitis during pregnancy.

**Figure 2:**
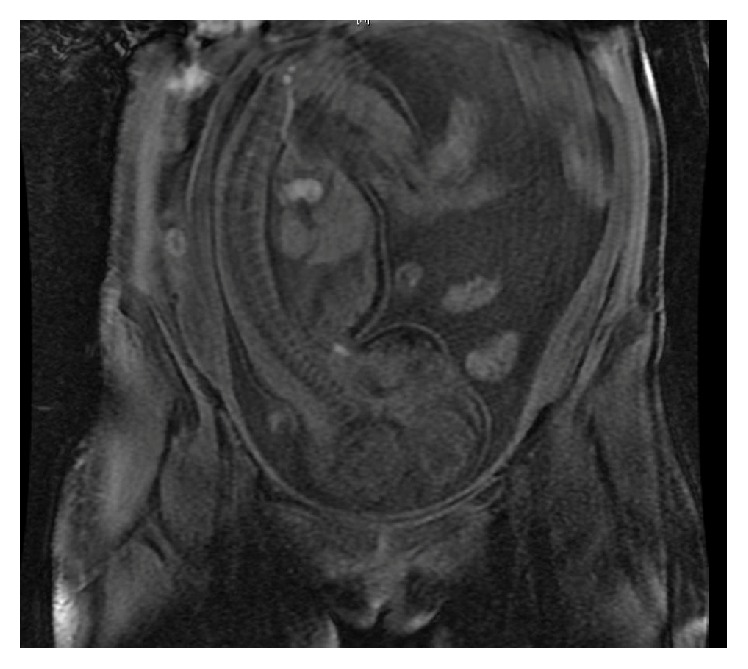
MRI showing coronal view of resolution of right iliacus muscle abscess following percutaneous drainage and antibiotics during pregnancy.

**Figure 3:**
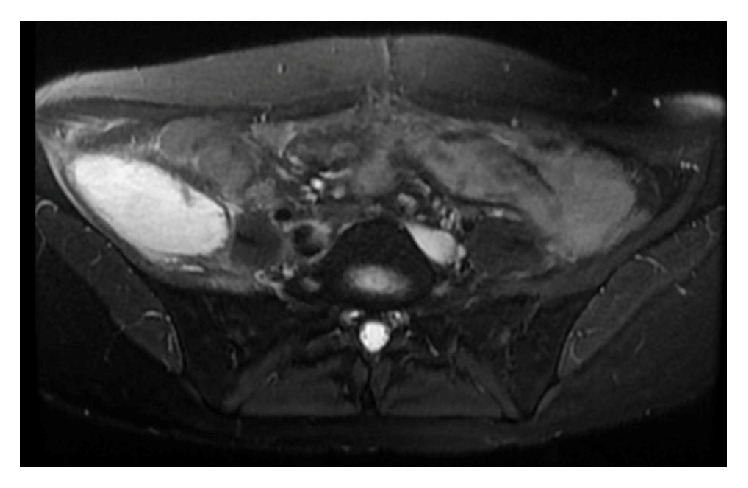
MRI showing axial view of right iliacus muscle sterile seroma.

**Figure 4:**
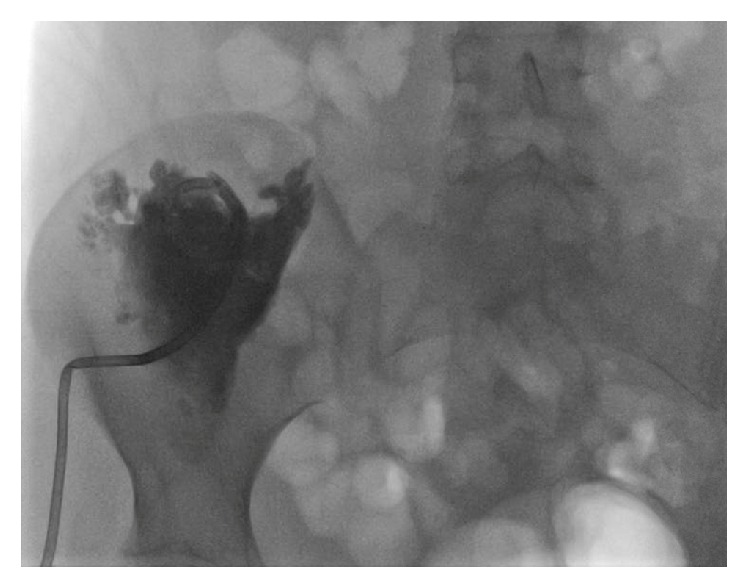
Abscessogram of the sterile seroma involving the right iliacus muscle, after drainage of septic abscess.

A 24-year-old female with a history of uncomplicated Crohn's ileitis since the age of 18 presented with right flank pain of 3 weeks' duration. She was 31 weeks pregnant. She had been in clinical remission on azathioprine maintenance therapy for her Crohn's ileitis. However, she elected to discontinue her azathioprine during her pregnancy. Physical examination was significant for tenderness in her right flank and limitation of range of motion due to pain in her right lower extremity. Magnetic resonance imaging (MRI) showed a right iliacus muscle abscess 7 × 5 cm (Figures 1(a) and 1(b)). Ultrasound-guided aspiration of the iliacus muscle abscess yielded 90 mL of purulent fluid. Cultures grew multiple enteric organisms. She responded to 4 weeks of intravenous antibiotics and percutaneous drainage, with resolution of the abscess by MRI (Figure 2). She delivered a healthy baby at 37 weeks of gestation by vaginal delivery after induction of labor. Two months postpartum, she complained of recurrence of her right flank discomfort. She had no fever or chills. Laboratory examination was normal, without leukocytosis. Computerized tomography revealed that the iliacus muscle fluid collection had recurred. There was no visible fistulous communication from the thickened ileum to the right iliacus muscle. Percutaneous aspiration revealed scattered white blood cells, but no organisms on gram stain, fungal stain, or culture. The sterile fluid collection was treated by percutaneous drainage until resolution, and then the drain was removed. She underwent ileal resection. No residual abscess or fistula was identified at surgery. One month postoperatively she again complained of right flank pain. She had no fever or leukocytosis. Imaging studies (Figure 3) revealed that the iliacus muscle fluid collection had again recurred. Percutaneous aspiration again revealed a sterile fluid collection. An abscessogram (Figure 4) outlined the seroma cavity and excluded an ongoing fistula. She responded to a prolonged course of percutaneous drainage and eventual sclerotherapy of the residual seroma cavity.

## 3. Discussion

We have described 2 cases of sterile seroma complicating drainage of purulent skeletal muscle abscess in Crohn's disease. In our first case, a sterile seroma occurred after surgical drainage of a chronic psoas muscle abscess [4]. In our current case, the sterile seroma developed after percutaneous drainage of an acute iliacus muscle abscess. Both cases occurred in the setting of Crohn's ileitis complicated by right-sided skeletal muscle septic abscesses. In our first case, the original psoas muscle abscess developed as a result of a documented fistula from Crohn's ileitis to the right psoas muscle. In our current case, no definite fistula was identified. However, the right iliacus muscle was contiguous to Crohn's ileitis. We therefore speculate that the patients' right iliacus purulent muscle abscess developed from an ileal fistula or from a microperforation of Crohn's ileitis with contiguous involvement of the right iliacus muscle. The ileal disease associated with the pyogenic skeletal muscle abscess was resected surgically in both of cases. Despite surgical resection of ileal Crohn's disease, and drainage of the skeletal muscle abscess, the fluid collection recurred.

Both patients presented after a previous successful drainage of a septic skeletal muscle abscess. The presenting symptom was discomfort, but without signs and symptoms of sepsis. Imaging studies revealed a fluid collection resembling the original septic pyogenic skeletal muscle abscess. Aspirates of the fluid collection were sterile. Imaging studies indicated that a fistula and/or perforation was no longer present. In both cases, the recurrent fluid collection was a sterile seroma. Treatment required prolonged percutaneous drainage, followed by sclerotherapy of the residual seroma cavity.

We postulate that the sterile seroma develops within the dead space carved out by the preceding septic skeletal muscle abscess. This mechanism of formation of sterile seromas differs from another rare entity termed aseptic abscesses [5–10] that can also occur in Crohn's disease in that the latter develops without a preceding septic process.

Sterile seromas require differentiation from purulent (septic) muscle abscesses, as their etiology, septic risk, and treatment differ. Awareness of this phenomenon is necessary to avoid confusion from a recurrent pyogenic skeletal muscle abscess.
